# Optimization of greywater treatment using UiO-66 nanomaterial: artificial neural network modeling

**DOI:** 10.1038/s41598-026-48174-2

**Published:** 2026-04-13

**Authors:** Aliakbar Zare, Mehrzad Feilizadeh, Zahra Derakhshan, Jamal Rasouli

**Affiliations:** 1 Department of Chemical Engineering, School of Chemical and Petroleum Engineering, Shiraz University, Shiraz, Iran; 2 Department of Environmental Health Engineering, School of Public Health, Shiraz University of Medical Sciences, Shiraz, Iran

**Keywords:** Adsorption process, Greywater, MOF, UiO-66, Wastewater treatment, ANN, Chemistry, Engineering, Environmental sciences, Materials science

## Abstract

Metal–organic frameworks (MOFs) have attracted considerable attention in wastewater treatment due to their high porosity and tunable structures. In this study, UiO-66 materials were synthesized using different molar ratios of zirconium tetrachloride to terephthalic acid (metal-to-ligand ratios) to evaluate their performance in greywater treatment. Structural and physicochemical properties of the synthesized materials were characterized using XRD, BET, FTIR, SEM, and EDS analyses. Among the prepared samples, UiO-66 with a metal-to-ligand ratio of 1:0.75 exhibited improved crystallinity, a higher surface area (1387 m²/g), and a larger total pore volume (1.66 cm³/g), indicating favorable structural properties for adsorption applications. Batch adsorption experiments were conducted to investigate the effects of initial COD concentration, adsorbent dosage, pH, and contact time on greywater treatment efficiency. To model the adsorption process and analyze the interactions between operational parameters, an artificial neural network (ANN) model was developed and combined with a genetic algorithm (GA) for process optimization. The model demonstrated high predictive capability and enabled the identification of optimal operating conditions, namely an initial COD concentration of 236 ppm, adsorbent dosage of 500 ppm, pH 2, and contact time of 3 h, yielding a maximum COD removal efficiency of 88.4%. Adsorption equilibrium analysis indicated that the Langmuir model provided the best fit to the experimental data, with a maximum adsorption capacity of 722 mg/g. Kinetic analysis revealed that the adsorption process followed a pseudo-second-order model. The results demonstrate that the optimized UiO-66 adsorbent exhibits promising potential for greywater treatment and that the combined experimental–modeling approach provides valuable insights for optimizing MOF-based adsorption processes.

**Supplementary Information:**

The online version contains supplementary material available at 10.1038/s41598-026-48174-2.

## Introduction

Amid growing concerns over water scarcity and environmental pollution^1^, greywater treatment has emerged as a strategic method to decrease freshwater demand and mitigate the ecological burden of wastewater discharge. Greywater, which constitutes approximately 50–80% of household effluents, is generated from activities such as bathing, laundry, and dishwashing, and is characterized by high levels of surfactants, oils, and biodegradable organic matter. Its substantial contribution to domestic wastewater volume, coupled with a relatively lower health risk compared to blackwater, makes it an ideal candidate for non-potable reuse applications such as landscape irrigation and cleaning^2,3^. However, greywater typically contains a wide range of organic contaminants, with reported chemical oxygen demand (COD) values varying from tens to several thousand ppm depending on household activities and greywater sources^4,5^. Accordingly, this study focuses on greywater as a representative and relevant target for wastewater treatment, with the aim of developing sustainable and high-efficiency adsorbents capable of addressing its complex and variable composition^6,7^.

Several methods have been developed for greywater treatment, including membrane filtration^8^, advanced oxidation processes (AOPs)^9^, adsorption and biological treatments^10^. For example, Harnessing light-driven chemical reactions offers a simple method for purifying water, as it can operate using both sunlight and common indoor lighting, making the needed energy source accessible in virtually all locations worldwide^11,12^.

Among them, adsorption is distinguished by its low cost, low energy demand, and capacity to attain high pollutant removal approaching up to 90% removal efficiency using relatively simple operating systems^13–17^. These advantages underline the urgency of developing advanced adsorbents tailored for greywater treatment applications^18,19^.

A wide range of studies have investigated different adsorbent materials for greywater treatment. Conventional adsorbents, including activated carbon and zeolites, have been extensively employed owing to their ready availability and high efficiency^20–22^. Recent advancements have highlighted the potential of MOFs in water treatment applications. Studies on MOFs like MIL-101, UiO-66, ZIF-8, ZIF-67, MIL-53, Ni-MOF, and HKUST-1, offering unique structural and functional advantages over traditional adsorbents^23^, have demonstrated high adsorption capacities for dyes and heavy metals, with efficiencies exceeding 90% in some cases. MOFs are highly porous, crystalline materials that can be engineered at the molecular level to achieve specific adsorption properties. Among the numerous MOFs synthesized to date, UiO-66, a zirconium-based framework, is particularly noteworthy for its high thermal and chemical stability, exceptional surface area, and robust framework integrity^24^. UiO-66 has demonstrated excellent capability for eliminating a range of contaminants, including heavy metals and organic pollutants, from aqueous solutions^25^. Wang et al.^26^ demonstrated that a Ce-UiO-66-NH₂@LS composite is able to adsorb up to 930.6 mg g⁻¹ of methyl orange and 282.8 mg g^− 1^ of hexavalent chromium at acidic pH, achieving removal efficiencies of 96.7% and 94.9% of initial pollutant concentration, respectively. Likewise, Xu et al.^27^ carried out a comprehensive evaluation of Zr-SA/Ce-UiO-66 in single- and binary-solute systems, showing that it eliminates 92% of initial phosphate concentration and the maximum adsorption capacity of the composite was determined to be 125 mg g⁻¹ at 313 K. Collectively, these findings highlight the considerable potential of MOFs for tackling a broad spectrum of organic and inorganic pollutants. Its acid resistance, for instance, enables effective operation at pH levels as low as 2, which is advantageous for treating greywater with varying pH conditions^28^. However, Challenges such as high synthesis costs, scalability, and stability under prolonged exposure to real-world water matrices remain to be addressed^29^. Nevertheless, UiO-66’s high adsorption capacity, tunable chemistry, and adaptability makes it an excellent candidate for greywater treatment, particularly for reducing COD content and other contaminants^30^.

The lack of research on UiO-66 for greywater treatment, combined with the need for advanced materials capable of addressing its diverse pollutant composition, underscores the importance of this research area. This study investigates controlled UiO-66 synthesis combined with data-driven modeling to systematically evaluate greywater treatment performance and optimize adsorption conditions, with a focus on understanding how synthesis conditions influence performance. UiO-66 adsorbents were synthesized using different molar ratios of precursors, and various characterization analyses were performed to evaluate their physicochemical properties. Additionally, the effects of key operational parameters—including adsorbent dosage, pH, initial COD concentration, and contact time—were assessed, and an artificial neural network (ANN) model integrated with a genetic algorithm (GA) was developed to analyze the relationships between operational variables and to identify optimal treatment conditions.

## Materials and methods

### Synthesis of UiO-66

**Fig. 1:**
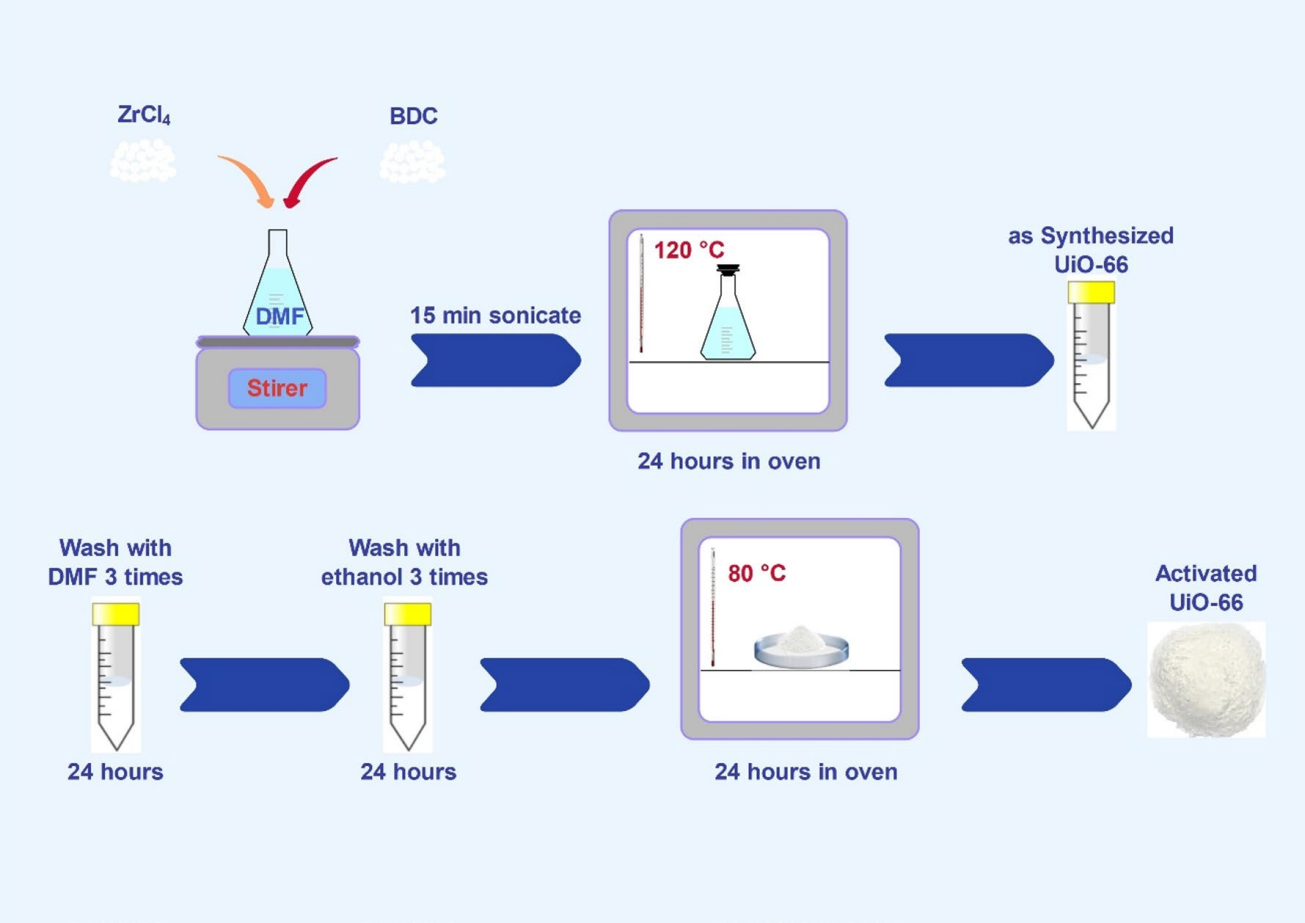
Schematic representation of the main stepsin UiO-66 metal–organic framework synthesis using terephthalic acid and zirconium tetrachloride as precursors in DMF solvent.

A complete list of materials can be found in the Supplementary Information. Three UiO-66 adsorbents with different molar ratios of zirconium tetrachloride to terephthalic acid namely 1:1, 1:0.75, and 1:0.5 were synthesized to evaluate the effect of synthesis ratio on structural and adsorption properties. The synthesis was performed using zirconium tetrachloride (ZrCl₄) as the metal precursor and benzene-1,4-dicarboxylic acid (BDC;terephthalic acid), as the organic linker/ligand. Dimethylformamide (DMF) was used as the solvent throughout the process. Initially, 30 mL of DMF was added to an Erlenmeyer flask and stirred gently. Then, 0.233 g of ZrCl₄ (1 mmol) was introduced and allowed to dissolve completely within five minutes. To achieve different metal-to-ligand molar ratios, the corresponding amount of BDC was added: 0.166 g (1 mmol) for UiO-66 1:1, 0.124 g (0.75 mmol) for UiO-66 1:0.75, and 0.083 g (0.5 mmol) for UiO-66 1:0.5. After the addition of BDC, the solution was sonicated for 15 min to ensure uniform mixing and then transferred to an oven where it was heated at 120 °C for 24 h to complete the reaction. The resulting suspension was centrifuged at 7000 rpm for 10 min to collect the synthesized product. The obtained solid was subjected to a washing procedure to remove residual reactants first washed with DMF for three cycles, followed by three cycles with ethanol. Each washing cycle included soaking the solid in the solvent for 24 h. The purified material was then dried in an oven at 80 °C for 24 h, weighed, and placed in labeled containers for later characterization and testing. The schematic of the main steps of the synthesis procedure is shown in Fig. 1.

### Characterization

The synthesized UiO-66 was examined using several analytical techniques to assess its structural and functional characteristics. X-ray diffraction (XRD) was used to identify the crystalline phase, employing a Philips PW1730 diffractometer with CuKα radiation (λ = 0.154 nm) over a 2θ range of 5–50°. Nitrogen adsorption–desorption measurements were carried out and interpreted via the Brunauer–Emmett–Teller (BET) method to determine the specific surface area, pore volume, and pore size distribution. The pore size distribution was further evaluated using the Barrett–Joyner–Halenda (BJH) model in combination with the Halsey equation. Surface morphology and elemental composition were investigated by scanning electron microscopy (SEM) and energy-dispersive X-ray spectroscopy (EDS) with elemental mapping. The functional groups present in the UiO-66 framework were identified by Fourier-transform infrared (FTIR) spectroscopy using a Varian Cary Eclipse instrument. Additionally, to evaluate structural stability and assess the potential for zirconium leaching under the experimental conditions, zirconium concentrations in the water samples were measured using graphite furnace atomic absorption spectrometry with the Thermo Scientific iCE 3500 Atomic Absorption Spectrometer.

### Design of experiments, modeling and optimization

The dataset used for artificial neural network modeling was generated from the Box–Behnken design (BBD). The BBD experimental matrix consisted of 17 runs, in which three operational parameters—initial COD concentration, adsorbent dosage, and solution pH—were varied to evaluate their effects on greywater pollutant removal efficiency. To investigate the effect of contact time, samples were taken from each run at 60, 120, and 180 min, giving a total of 51 data points based on the same BBD design. The experimental results obtained from this design provided a structured dataset that captures both the individual and interaction effects of the variables. These experimentally obtained data were subsequently used as input–output pairs for training and validating the ANN model. To effectively simulate and optimize greywater adsorption using UiO-66–based adsorbents, a feed-forward artificial neural networks constructed because of its strong ability to learn complex, nonlinear interactions among multiple variables. The ANN consisted of three layers: an input layer with four neurons corresponding to the main operating factors (initial COD, adsorbent dosage, pH, and contact time), a hidden layer with an optimized number of neurons, and a single-neuron output layer representing adsorption efficiency. The model was developed in MATLAB R2018b, and the Levenberg–Marquardt back-propagation algorithm was employed to iteratively update weights and biases during training. For robust prediction, the dataset was randomly divided into training (70%), validation (15%), and testing (15%) subsets. A hyperbolic tangent sigmoid function was used as the activation function in the hidden layer, while linear transfer functions were adopted for the input and output layers. Different network configurations were examined by adjusting the number of hidden neurons between 2 and 10, and each architecture was run ten times to minimize the influence of random weight initialization and to ensure result reproducibility. The predictive quality of each network was quantified using several statistical indicators—coefficient of determination (R²), adjusted R², root mean square error (RMSE), mean absolute error (MAE), and average absolute deviation (AAD)—calculated according to Eqs. 1–5, enabling reliable comparison and selection of the optimal model^31,32^.

1 $$\:{R}^{2}\:=\:1-\frac{\sum\:_{i=1}^{n}{\left({y}_{i,\:cal}-{y}_{i,\:exp}\right)}^{2}}{\sum\:_{i=1}^{n}{\left({y}_{ave,\:exp}-{y}_{i,\:exp}\right)}^{2}}$$

2 $$\:{R}_{adj}^{2}\:=\:1-\left[\frac{\left(1-{R}^{2}\right)\left(n-1\right)}{n-K-1}\right]$$

3 $$\:RMSE\:=\:\sqrt{\frac{\sum\:_{i=1}^{n}{\left({y}_{i,\:cal}-{y}_{i,\:exp}\right)}^{2}}{n}}$$

4 $$\:MAE\:=\:\frac{\sum\:_{i=1}^{n}\left|{y}_{i,\:cal}-{y}_{i,\:exp}\right|}{n}$$

5 $$\:AAD\:=\:\frac{1}{n}{\sum\:}_{i=1}^{n}\frac{\left|{y}_{i,\:cal}-{y}_{i,\:exp}\right|}{{y}_{i,\:exp}}\times\:100$$

Here, y_,cal_, y_,exp_, and y_ave, exp_ represent the predicted values, the observed experimental values, and the mean of all collected experimental data, respectively. The symbol n indicates the total number of data points, and K denotes the number of independent input parameters used in the model.

To optimize process conditions, the trained ANN model was integrated with a genetic algorithm (GA), a stochastic, population-based search method known for its efficiency in handling nonlinear optimization problems. The GA began with a population of 50 randomly generated individuals, with stochastic universal sampling as the selection function and rank-based scaling as the fitness function. Each generation produced new solutions through 80% crossover and 15% mutation, while 5% of the top-performing individuals (elites) were retained to maintain strong genetic material. Scattered crossover and Gaussian mutation operators were used to introduce diversity and ensure convergence. The trained ANN served as the fitness function in the GA framework, enabling the identification of optimal values for initial COD, pH, adsorbent concentration, and contact time that maximize greywater pollutant removal. To further interpret the ANN model, Garson’s algorithm (Eq. 6) was employed to determine the relative importance of each input variable. This analysis quantified the contribution of each factor to the overall adsorption efficiency by evaluating the absolute weights of connections between layers^33,34^.

6 $$\:{I}_{j}=\:\frac{{\sum\:}_{m=1}^{m={N}_{h}}\left(\right(\raisebox{1ex}{$\left|{W}_{jm}^{ih}\right|$}\!\left/\:\!\raisebox{-1ex}{${\sum\:}_{k=1}^{k={N}_{i}}\left|{W}_{km}^{ih}\right|$}\right.)\times\:\left|{W}_{mn}^{ho}\right|)}{{\sum\:}_{k=1}^{k={N}_{i}}\left\{{\sum\:}_{m=1}^{m={N}_{h}}\left(\right(\raisebox{1ex}{$\left|{W}_{km}^{ih}\right|$}\!\left/\:\!\raisebox{-1ex}{${\sum\:}_{k=1}^{k={N}_{i}}\left|{W}_{km}^{ih}\right|$}\right.)\times\:\left|{W}_{mn}^{ho}\right|)\right\}}$$

Where Iⱼ is the relative importance of the jᵗʰ input, W represents the connection weights, W_i_ₘ is the weight from input neuron i to hidden neuron m, and Wₘₒ is the weight from hidden neuron m to output neuron o.

### Greywater removal experiments

Adsorption experiments were performed under strictly controlled conditions to assess the efficacy of UiO-66 in chemical-oxygen-demand (COD) reduction. 200 mL of synthetic greywater (the composition is provided in Table S1) was prepared at predetermined COD levels (200–400 ppm) and the pH was subsequently adjusted to the desired value. A measured mass of UiO-66 was dispersed in the solution by 15 min sonication, ensuring homogeneous contact between adsorbent and contaminants. The suspension was agitated for 3 h, and 5-mL aliquots were withdrawn at 60-min intervals. Each aliquot was clarified by centrifugation (8000 rpm, 15 min) prior to COD analysis. COD was quantified spectrophotometrically using the closed-reflux colorimetric method. Calibration standards ranging from 100 to 500 ppm were prepared from a 500 ppm potassium hydrogen phthalate stock solution (0.425 g L⁻¹) via serial dilution. For every measurement, 2.5 mL of sample or standard was transferred to a sealed COD vial, digested at 150 °C for 2 h, cooled to room temperature, and analyzed at 600 nm. The resulting absorbance values were converted to COD concentrations using the calibration curve (see Figure S1).

### Kinetic and equilibrium study

Batch adsorption experiments were conducted to evaluate the adsorption kinetics and equilibrium behavior of greywater pollutants onto the synthesized UiO-66 adsorbents. For the kinetic studies, a fixed amount of adsorbent was added to greywater solutions with a known initial COD concentration under controlled conditions. The mixtures were agitated at constant temperature, and samples were withdrawn at predetermined time intervals to determine the residual COD concentration. The adsorption capacity at time (q_t_) was calculated using the measured COD values to analyze the adsorption rate and mechanism. The experimental kinetic data were subsequently fitted using pseudo-first-order PFO (Eq. 7) and pseudo-second-order PSO (Eq. 8) kinetic models. For the adsorption isotherm studies, a series of batch experiments were performed using different initial COD concentrations while maintaining constant adsorbent dosage, pH, and contact time sufficient to reach adsorption equilibrium. After equilibrium was achieved, the remaining COD concentration in the solution was measured and used to calculate the equilibrium adsorption capacity (q_e_). The equilibrium data were then analyzed using Langmuir, Freundlich, and Temkin (Eqs. 9–11) isotherm models to evaluate the adsorption characteristics and interactions between the adsorbent surface and the organic pollutants present in greywater.

7 $$\:{q}_{t}={q}_{e}(1-{e}^{-{k}_{1}t})\:\:\:\:PFO$$

8 $$\:{q}_{t}=\frac{{q}_{e}^{2}{K}_{2}t}{1+{k}_{2}{q}_{e}t}\:\:\:\:PSO$$

9 $$\:{q}_{e}=\frac{{q}_{m}{K}_{L}{C}_{e}}{(1+{K}_{L}{C}_{e})}\:\:\:\:Langmuir$$

10 $$\:{q}_{e}={K}_{F}{C}_{e}^{\frac{1}{n}}\:\:\:Freundlich$$

11 $$\:{q}_{e}=B*\mathrm{l}\mathrm{n}\left({K}_{T}{C}_{e}\right)\:\:\:\:Temkin$$

In these equations, qₜ (mg g⁻¹) is the adsorption capacity at time t (min), qₑ (mg g⁻¹) represents the equilibrium adsorption capacity, k₁ (min⁻¹) is the pseudo-first-order rate constant, k₂ (g mg⁻¹ min⁻¹) is the pseudo-second-order rate constant, qₘ (mg g⁻¹) is the maximum adsorption capacity, K_L_ (L mg⁻¹) is the Langmuir equilibrium constant related to adsorption affinity, Cₑ (mg L⁻¹) is the equilibrium COD concentration after adsorption, KF ((mg g⁻¹) (L mg⁻¹) (1/n)) is the Freundlich adsorption constant, n is the adsorption intensity parameter, B (J mol⁻¹) is a constant related to the heat of adsorption and, K_T_ (L mg⁻¹) is the Temkin equilibrium constant.

## Result and discussion

The performance of the three synthesized samples (i.e., UiO-66 1:1, UiO-66 1:0.75, and UiO-66 1:0.5) was compared in terms of COD removal from greywater, and the results are shown in Figure S2. As presented in this figure, UiO-66 1:1 and UiO-66 1:0.75 exhibited superior performance for greywater treatment. Accordingly, characterization analyses are provided in the following section for only these two samples.

### Materials characterization

**Fig. 2:**
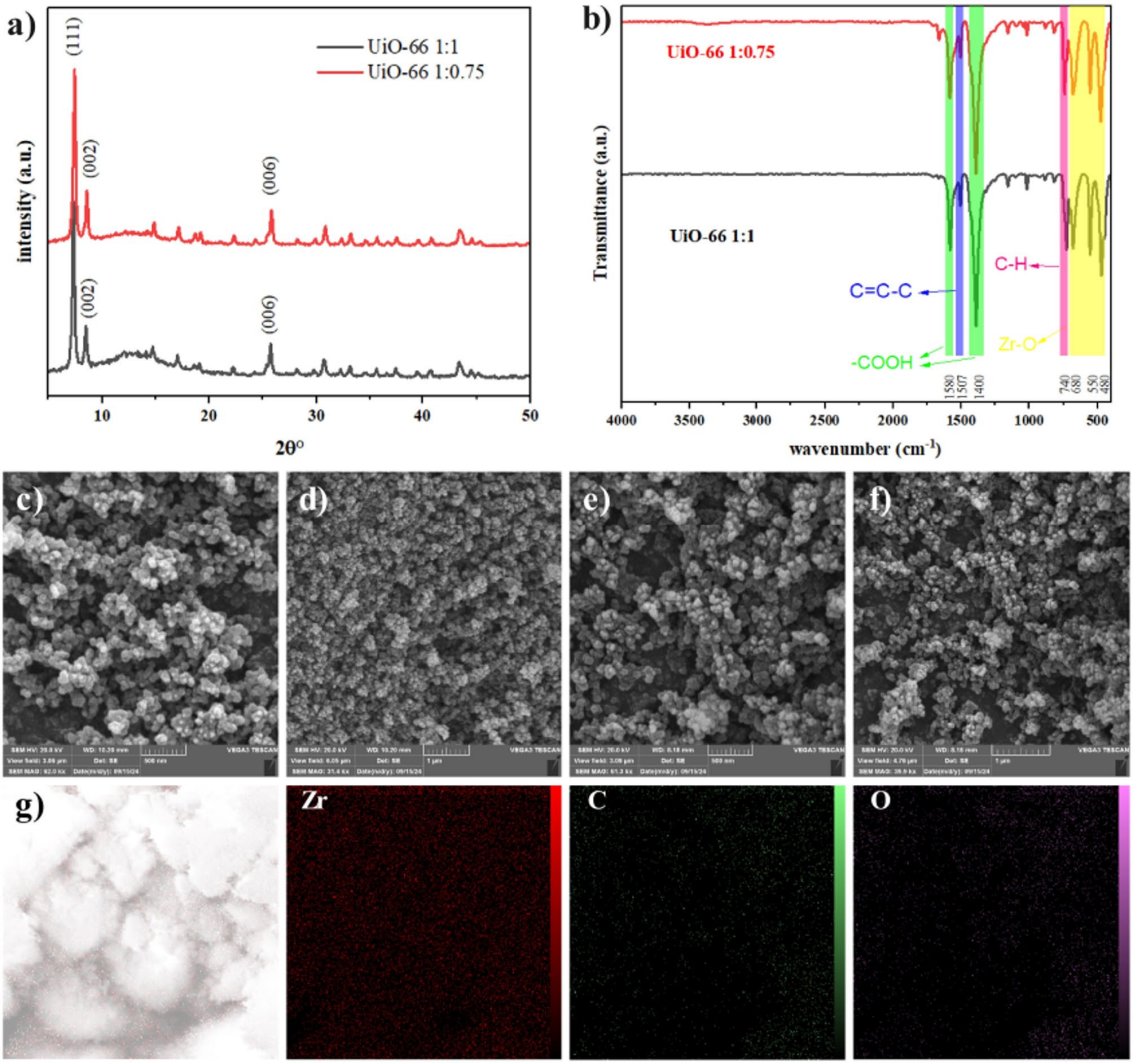
(**a**) XRD patterns of UiO-66 synthesized with molar ratios of 1:1 and 1:0.75, (**b**) FTIR of the UiO-66 samples, color codes: green: -COOH, purple: C = C-C, pink: C-H and yellow: Zr-O. (**c** and **d**) SEM image of UiO-66 synthesized at a 1:1 precursor molar ratio, (**e** and **f**) SEM image of UiO-66 synthesized at a 1:0.75 precursor molar ratio, (**g**) elemental mapping of UiO-66 1:0.75, illustrating the homogeneous distribution of Zr, C, and O within the framework.

In the XRD patterns shown in Fig. 2a, both UiO-66 samples (1:1 and 1:0.75) exhibit the characteristic diffraction peaks that are commonly associated with the UiO-66 crystalline structure. These include prominent reflections around 2θ = 7.3°, 8.5°, 25.7°, 30.7°, and 43.3°, which can be indexed to the (111), (200), (220), (400), and (331), and higher-order lattice planes, respectively (JCPDS- 4512072)^35,36^. The UiO-66 1:0.75 sample, however, displays more intense and sharper peaks compared to its 1:1 counterpart, indicating improved crystallinity and fewer structural defects. Specifically, the main diffraction peak near 7.3° (111) appears more pronounced, and the reflections at 8.5° (200) and 25.7° (220) are sharper, suggesting that the lower terephthalic acid to zirconium ratio led to better-ordered zirconium clusters and a more uniform incorporation of organic linkers. The relative intensity enhancements in UiO-66 1:0.75’s pattern, as well as the reduced peak broadening, provide strong evidence that tuning the metal-to-linker ratio results in a more homogeneous and stable framework^36^. Such an improvement in crystallinity is scientifically significant, as well-ordered MOF structures often yield greater porosity, more accessible adsorption sites, and enhanced chemical stability, thereby making UiO-66 1:0.75 a superior candidate for adsorption-based applications than the 1:1 variant.

Turning to the FTIR spectra displayed in Fig. 2b, both samples show the characteristic vibrations associated with the terephthalate linkers and the Zr–O clusters of UiO-66. The presence of intense bands around 1570 and 1390 cm⁻¹ can be attributed to the asymmetrical and symmetrical stretching vibrations of the carboxylate groups, while the aromatic C–C stretching modes of the benzene ring appear between 1500 and 1600 cm⁻¹^37^. Both samples also exhibit weaker signals corresponding to C–H out-of-plane bending modes in the range of 700–900 cm⁻¹, and broad bands below 700 cm⁻¹ that can be linked to the Zr–O–C bonds forming the metal–organic nodes^38^. Nevertheless, a careful comparison reveals that the UiO-66 1:0.75 spectrum shows slightly more defined carboxylate-related peaks with better-resolved intensity ratios, suggesting that the altered synthesis conditions facilitated a more uniform coordination environment between the zirconium clusters and the carboxylate ligands. This indicates a lower degree of defect sites and a more effective incorporation of the organic linker in the 1:0.75 sample. Such finer spectral features are scientifically meaningful because a purer, more intact coordination environment often correlates with improved mechanical robustness and chemical resilience.

Examining the SEM micrographs for UiO-66 1:1 and 1:0.75, it is evident that both samples form aggregates composed of nanosized crystallites (Fig. 2c-f). However, the UiO-66 1:0.75 image reveals a more uniform particle size distribution and a more cohesive arrangement of crystallites, which appear as well-defined, relatively spherical aggregates. In contrast, the 1:1 sample tends to display a slightly less uniform morphology, with broader particle size distributions and a less orderly assembly of the MOF crystals. The improved homogeneity observed in UiO-66 1:0.75 can be tied back to the more optimal synthesis ratio, resulting in a nucleation and growth process that yields consistent crystal domains. From a scientific standpoint, a more uniform particle morphology often translates into greater accessibility of the pore network, improved surface area utilization, and more predictable mass transfer properties, thereby enhancing adsorption performance and stability under various operational conditions.

The EDS spectra presented in Figures S3a and S3b provide further confirmation of the successful synthesis and elemental composition of the UiO-66 adsorbents prepared with different precursor ratios. The spectra clearly show the characteristic peaks corresponding to carbon (C), oxygen (O), and zirconium (Zr), which represent the primary elements forming the UiO-66 framework. The absence of additional elemental peaks indicates that no significant impurities or secondary phases were introduced during the synthesis process. For the UiO-66 1:0.75 sample, the quantitative elemental analysis reveals weight percentages of 40.57% C, 33.71% O, and 25.72% Zr. These values are in good agreement with the expected composition of the UiO-66 structure, confirming that the organic terephthalate linkers and zirconium clusters were successfully incorporated into the framework. The balanced distribution of these elements suggests that the optimized precursor ratio promotes a more favorable framework formation, leading to a well-structured MOF with improved structural integrity. Similarly, the UiO-66 1:1 sample exhibits elemental weight percentages of 42.62% C, 33.52% O, and 23.86% Zr. Although both materials display similar elemental compositions consistent with the UiO-66 framework, the slight differences in elemental ratios may reflect variations in the coordination environment and framework organization resulting from the different synthesis ratios. Overall, the EDS results support the successful formation of zirconium-based MOF structures in both samples and confirm the effective integration of the organic linker and metal nodes within the framework.

**Fig. 3:**
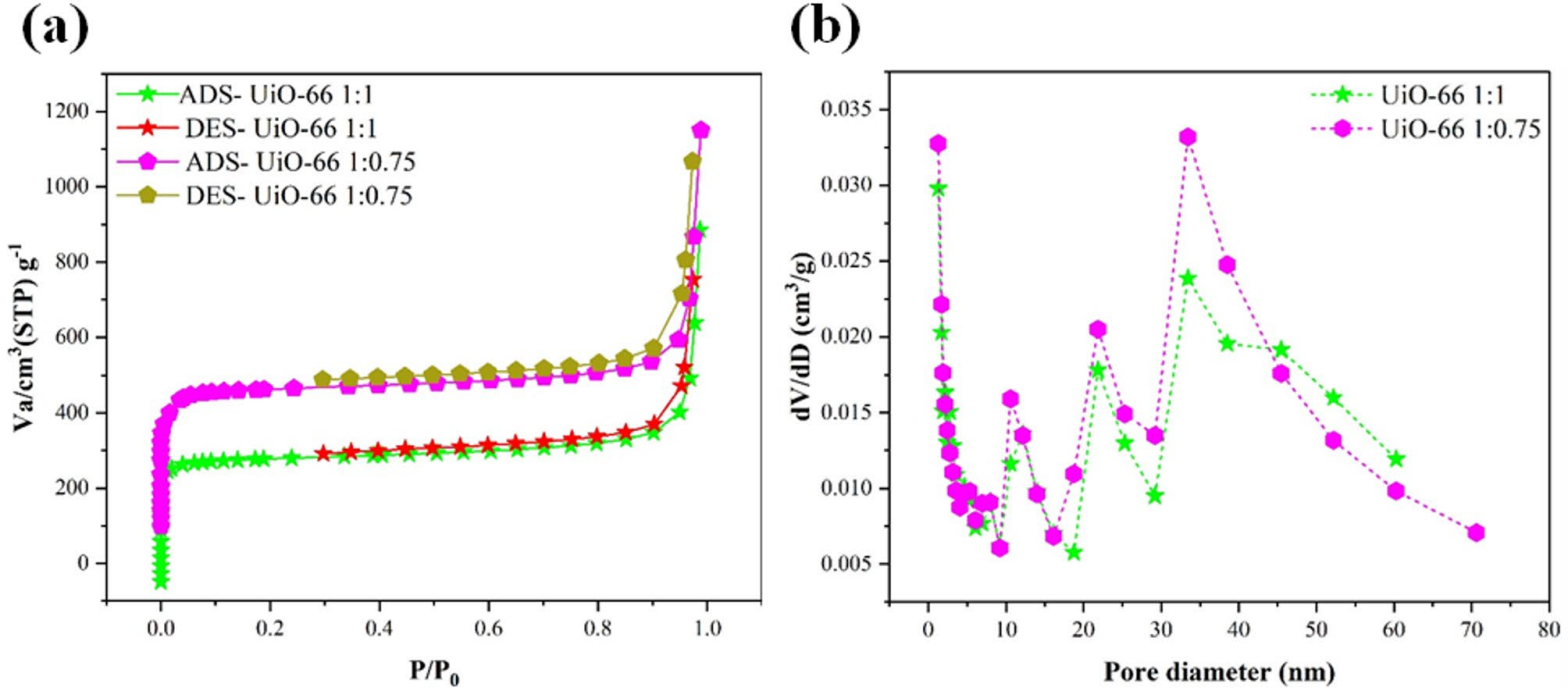
(**a**) N_2_ adsorption and desorption isotherms and (**b**) the corresponding pore size distribution curve of UiO-661:1 and UiO-66 1:0.75.

The N₂ adsorption-desorption isotherms presented in Fig. 3a align with a Type IV behavior according to the IUPAC classification, reflecting the presence of both microporosity and mesoporosity in the UiO-66 frameworks. At low relative pressures, the steep uptake in both UiO-66 samples is typical of microporous materials, as nitrogen molecules readily fill the abundant micropores. As the pressure increases, the occurrence of a pronounced hysteresis loop at intermediate to high relative pressures indicates the presence of mesopores and larger pore cavities. The shape of this hysteresis loop suggests a Type H3 classification, which is often associated with narrow slit-like pores or pore structures formed by aggregates of small particles, as is commonly observed in defect-engineered MOFs. The UiO-66 1:0.75 sample, in particular, exhibits a more pronounced hysteresis loop and greater nitrogen uptake at higher relative pressures, confirming the formation of additional and more accessible mesopores compared to the 1:1 variant. Moreover, this textural enhancement is substantiated by the quantitative data summarized in Table 1, which shows that UiO-66 1:0.75 possesses a higher BET surface area (1387 m²/g) than UiO-66 1:1 (1257 m²/g). Additionally, the 1:0.75 sample demonstrates an increased total pore volume (1.66 cm³/g versus 1.48 cm³/g for the 1:1 variant), indicating a more substantial network of interconnected pores capable of accommodating greater amounts of adsorbate. The slightly larger average pore diameter (4.79 nm for 1:0.75 versus 4.71 nm for 1:1) further points to a modest shift towards mesopore formation and enhanced structural openness^39^. These combined improvements not only reflect the superior textural properties of UiO-66 1:0.75 but also have practical implications, as higher surface area and more accessible pore systems can facilitate faster diffusion and more efficient adsorption of target contaminants^40^. Figure 3b presents the BJH pore size distribution curves for UiO-66 1:1 and UiO-66 1:0.75. UiO-66 1:0.75 displays a broader and more intense distribution between 10 and 40 nm, indicating a more heterogeneous and accessible pore structure^41^. This aligns with its higher BET surface area and pore volume, suggesting enhanced structural openness. In contrast, the narrower distribution in UiO-66 1:1 implies a more compact pore network. These results confirm that adjusting the metal-to-ligand ratio influences pore architecture, with the 1:0.75 sample exhibiting superior textural characteristics favorable for adsorption applications^42^.

**Table 1:** Comparison of surface area, pore volume, and average pore diameter for UiO-66 1:1 and UiO-66 1:0.75 samples.

Sample	BET surface area (m^2^/g)	Pore volume (cm^3^/g)	Pore diameter (nm)
UiO-66 1:1	1257	1.48	4.71
UiO-66 1:0.75	1387	1.66	4.79

Overall, UiO-66 1:0.75 exhibits significantly higher removal efficiency throughout the adsorption period compared to the other variants (see Figure S2), which can be attributed to its improved crystallinity, better-defined adsorption sites, uniform framework morphology, and enhanced surface area. Therefore, further studies on greywater treatment using UiO-66 are presented in the following sections for only the 1:0.75 ratio.

### ANN model

Table S2 presents the results of greywater removal efficiency as a function of the experimental design, showing variations in the initial COD, adsorbent concentration, pH, and adsorption time. These results reflect the individual and interaction effects of these parameters on removal efficiency, as predicted by the ANN. The results indicate a wide range of removal efficiencies, from approximately 32% to 86%, highlighting the significant influence of the studied parameters.

**Fig. 4:**
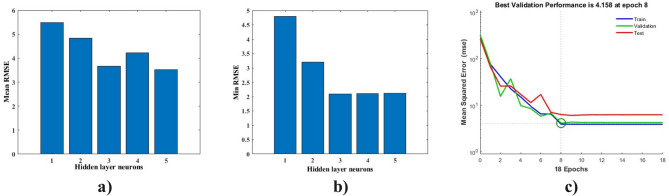
(**a**) Average RMSE in terms of the number of hidden layer neurons, (**b**) Minimum RMSE in terms of the number of hidden layer neurons, (**c**) Optimal number of epochs based on MSE.

Figure 4a illustrates the mean root mean square error (RMSE) across varying numbers of hidden neurons in the ANN model, revealing a decreasing trend in RMSE as the number of neurons increases, followed by a plateau. This behavior indicates that increasing neurons initially improves model performance by capturing more intricate relationships, but additional neurons provide diminishing returns.

Figure 4b shows the minimum RMSE for the different hidden neuron configurations, identifying the configuration with the lowest error. The results suggest that an optimal number of neurons balances model complexity and computational efficiency while avoiding overfitting^43^.

Figure 4c highlights the convergence of the ANN during training. The mean squared error (MSE) decreases rapidly in the initial epochs and stabilizes after the 8th epoch, indicating that the model reaches its optimal performance early. Moreover, it demonstrates that the best validation performance, with an MSE of 4.158, occurs at the 8th epoch, further validating the ANN’s effective training process and robustness. Finally, overtraining is avoided by selecting this optimal epoch point, ensuring the model generalizes well to unseen data. Figure S4b presents a scatter plot comparing laboratory-measured adsorption efficiencies with ANN-predicted values. The tight clustering of data points around the diagonal (perfect agreement line) indicates that the ANN achieves high predictive accuracy. The close correspondence confirms the model’s ability to replicate real-world adsorption outcomes reliably. The schematic of the ANN model used is presented in Figure S5.

The statistical adequacy of the ANN model was further evaluated using several fit statistics summarized in Table S3. The coefficient of determination (R² = 0.983) demonstrates that the developed model explains approximately 98.3% of the variability in greywater removal efficiency, indicating a strong agreement between predicted and experimental values. Similarly, the adjusted R² value (0.982) is in close agreement with the values of training R² (0.986), testing R² (0.971), and validation R² (0.979), suggesting that the model has good predictive capability without significant overfitting. Additionally, other statistical parameters such as RMSE, MSE, MAE and AAD of the ANN model are calculated and their values are 2.117, 4.480, 1.436 and 2.130 respectively. Overall, these statistical indicators confirm the robustness and suitability of the ANN model for describing the effects of the investigated parameters on adsorption performance.

### Effect of parameters

**Fig. 5:**
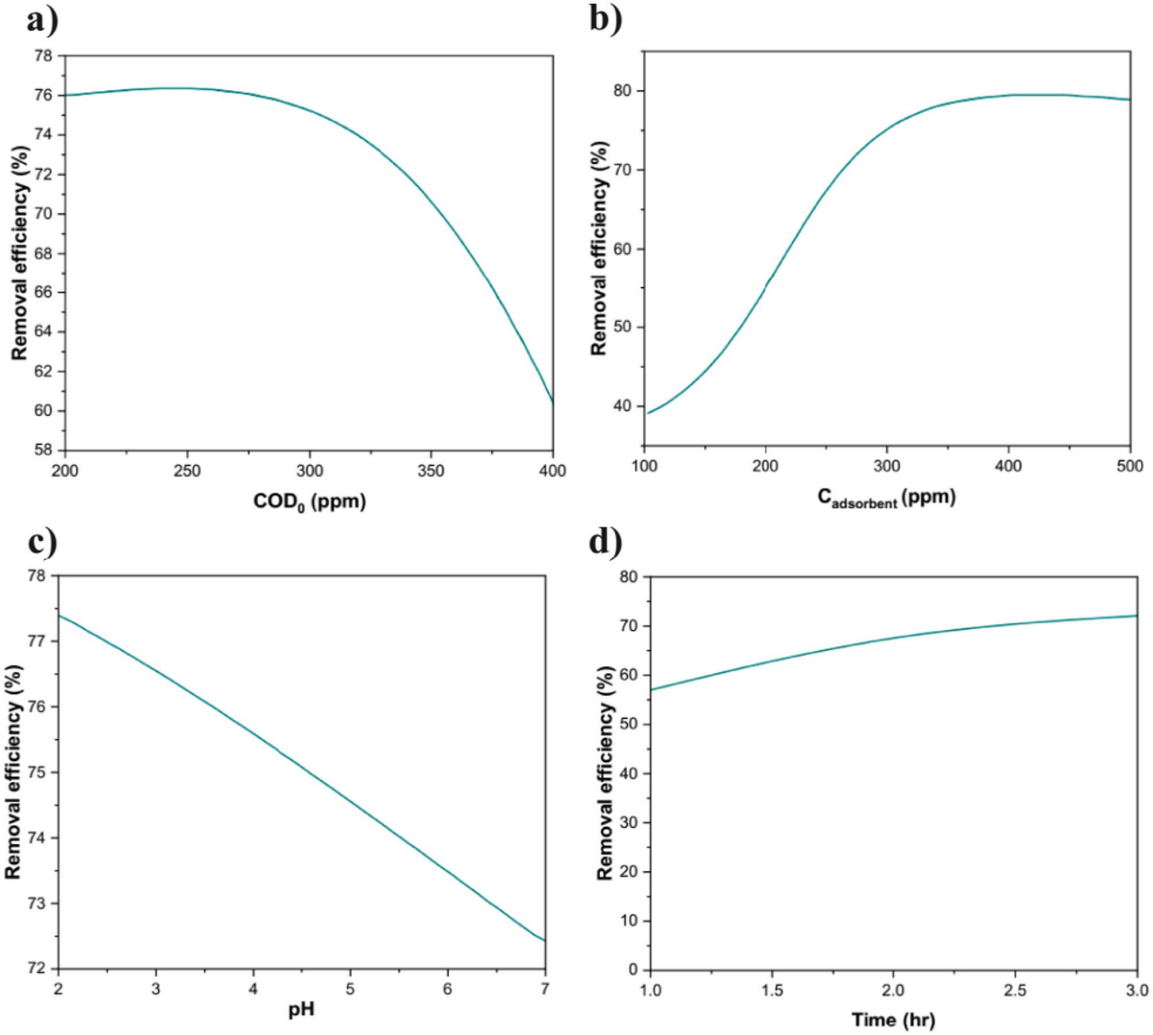
(**a**) effect of initial COD, (**b**) effect of adsorbent concentration, (**c**) effect of pH and (**d**) effect of time on the COD removal efficiency of greywater using UiO-66 1:0.75.

The removal efficiency of greywater using UiO-66 1:0.75 was systematically evaluated and modeled as a function of key operational parameters, including initial COD, adsorbent concentration, pH, and adsorption time. The impact of the COD_0_ on removal efficiency is presented in Fig. 5a. The results demonstrate a non-linear trend, where removal efficiency increases with rising COD levels before declining beyond an optimal point. This behavior is attributed to the interaction between pollutant molecules and the active sites on the adsorbent surface. At lower COD concentrations, the active sites on the UiO-66 framework are readily available, leading to efficient adsorption. However, as the COD_0_ increases, the adsorbent surface becomes progressively saturated, and removal efficiency declines due to a limitation in active site availability^44^. The initial increase in efficiency is likely driven by a higher mass transfer driving force at elevated COD levels, while the subsequent saturation effect is consistent with Langmuir-type adsorption isotherms (presented in Sect.  3.5), in which the finite number of adsorption sites limits the removal capacity at high pollutant concentrations^44^.

Figure 5b highlights the relationship between adsorbent concentration and removal efficiency. A clear positive correlation is observed, indicating that increasing the UiO-66 1:0.75 dosage enhances pollutant removal due to the greater availability of active sites and surface area. However, at excessively high concentrations, the efficiency gain may plateau or even slightly decline. This behavior can be attributed to particle aggregation or caking, which reduces the effective surface area and hinders pollutant diffusion, thereby limiting additional adsorption. Such findings emphasize the importance of optimizing adsorbent dosage to maximize efficiency without inducing physical limitations or unnecessary material use^45^.

The effect of pH on removal efficiency, depicted in Fig. 5c, reveals that acidic conditions are more favorable for greywater treatment using UiO-66 1:0.75. A decreasing trend in removal efficiency with increasing pH indicates that lower pH values enhance adsorption. This may be attributed to the protonation of the adsorbent surface under acidic conditions (pHpzc = 6.4; Figure S4a). The synthetic greywater contains anionic species such as phosphate (from Na₂PO₄), sulfate (Na₂SO₄), and possible anionic surfactants (from laundry detergent, shampoo, soap ~ 18 g total), and it had an initial pH of 4.8. At pH < 6.4, UiO-66 exhibits positive surface charge, enabling attraction to these negatively charged pollutants. As a result, although other mechanisms may also contribute to enhanced adsorption, this surface charge modification can promote electrostatic interactions with negatively charged or polar organic pollutants^46,47^. In contrast, higher pH values result in deprotonation of the UiO-66 surface, weakening these interactions and reducing the adsorbent’s effectiveness^48,49^. More detail is provided in Sect.  4 of the Supplementary Information.

Figure 5d illustrates the effect of reaction time on removal efficiency during the adsorption process. This figure depicts the predicted response in removal efficiency from 60 to 180 min into the experiment. As shown, removal efficiency increases steadily with time, indicating the progressive occupation of adsorption sites as equilibrium is approached. The initial increase in removal efficiency (~ 57%) occurs during the first hour (0 to 60 min). This initial increase is driven by the abundance of unoccupied active sites, facilitating a high adsorption rate. Over time, as these sites become occupied, the adsorption rate slows, eventually leading to a plateau as equilibrium is established^50^.

**Fig. 6:**
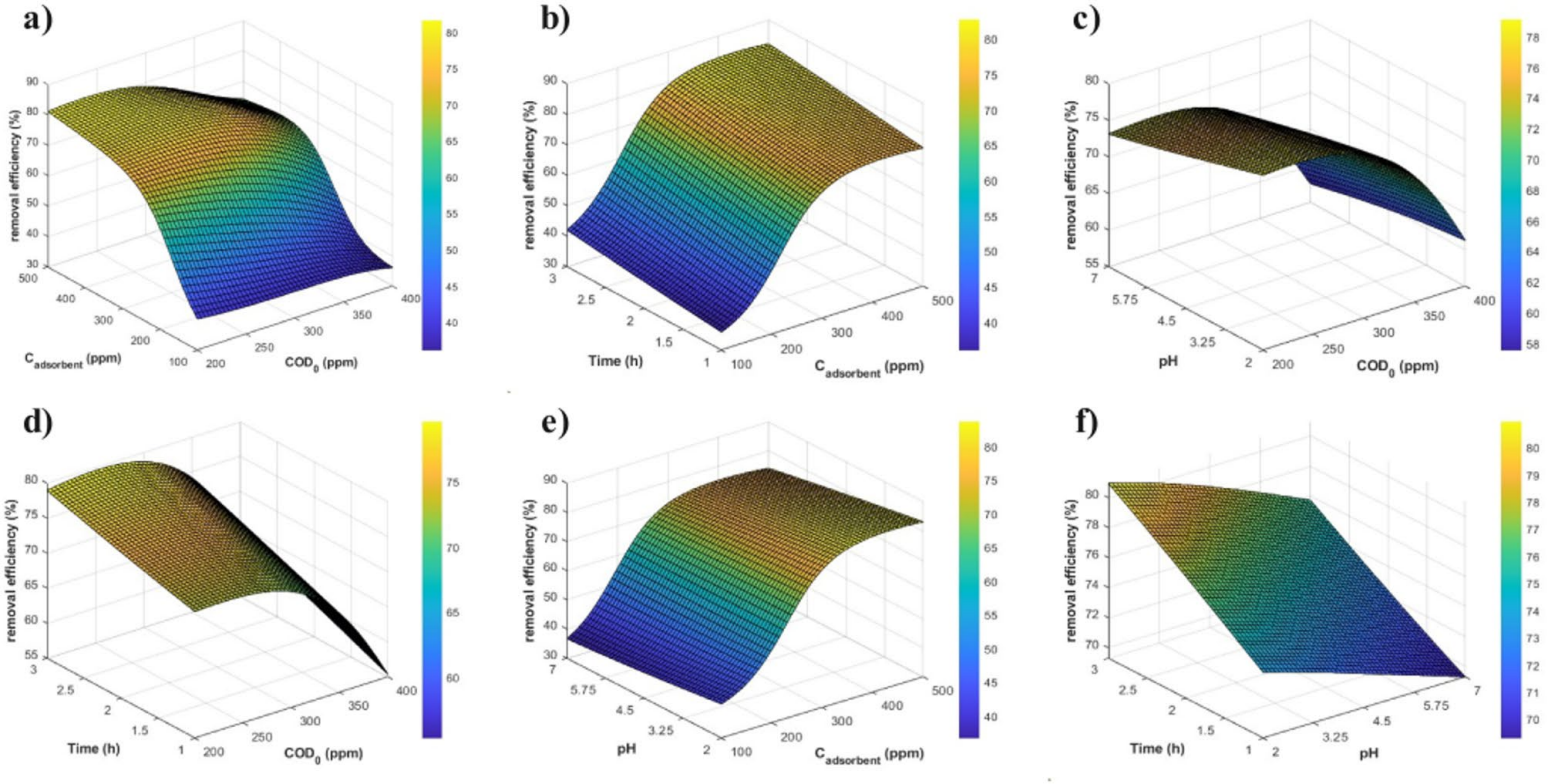
3D plots showing the effect of parameters on COD removal: (**a**) C_adsorbent_ and COD_0_, (**b**) Time and C_adsorbent_, (**c**) pH and COD_0_, (**d**) Time and COD_0_, (**e**) pH and C_Adsorbent_, and (**f**) Time and pH.

Figures 6a–f display three-dimensional response-surface plots that elucidate how key operating variables interact to influence COD removal from greywater using the optimized UiO-66 1:0.75. The discussion and analysis of these plots are provided in Sect.  4 of the Supplementary Information.

### Optimization and relative importance of parameters

The genetic algorithm was employed to identify the optimal conditions for greywater treatment, maximizing removal efficiency while optimizing the operational feasibility of the process. The optimized parameters and their corresponding values are summarized in Table S4. The results indicate that the optimal COD_0_ is 236 ppm, with an adsorbent concentration of 500 ppm, a pH of 2, and an adsorption time of 3 h. Under these conditions, the removal efficiency reaches a maximum of 88.4%. These values are fully consistent with the parameter trends discussed above, confirming the validity of the optimization.

Garson’s method was used to determine the relative influence of each input parameter. According to the results of this method (presented in Table S5), the relative importance values for initial COD concentration, pH, and contact time were 33%, 11.5%, and 7.6%, respectively. Therefore, the adsorbent dosage was the most effective parameter for COD removal from greywater, accounting for 47.9% relative importance. This is evident in Fig. 5b, where increasing the adsorbent dosage led to a rapid increase in COD removal efficiency.

### Adsorption isotherms and kinetic

**Fig. 7:**
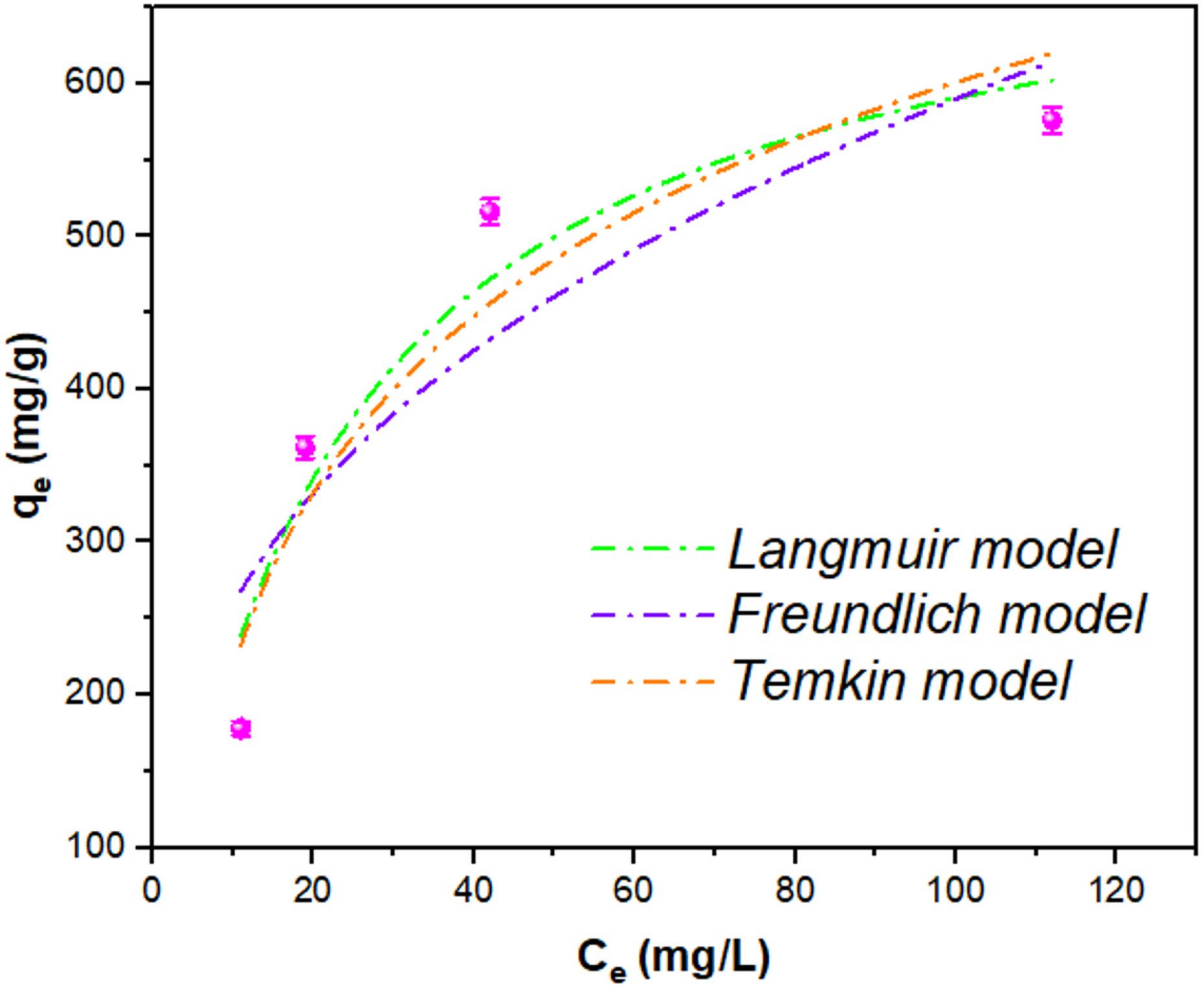
Adsorption isotherms of Langmuir, Freundlich and Temkin models.

Isotherm adsorption models play a crucial role in elucidating the interactions between adsorbents and contaminants in greywater, providing key insight into adsorption capacity and efficiency. Among the frequently applied models, Freundlich, Langmuir, and Temkin -each describes the adsorption mechanism from a different theoretical standpoint- are presented here. In this work, the experimental adsorption data were fitted to all three models. The adsorption isotherms of these three models are shown in Fig. 7, while the model parameters are presented in Table 2. According to the table, the Langmuir isotherm exhibited the best fit (R² = 0.924) indicating monolayer adsorption consistent with the numerous micropores in the UiO-66 structure (confirmed by N₂ adsorption/desorption analysis). Adsorption thus occurs as a monolayer within these micropores, yielding a maximum capacity (qₘ) of 722 mg/g. This exceptionally high capacity underscores the excellent performance of the UiO-66 1:0.75 adsorbent for greywater pollutant removal^51,52^. The Freundlich model showed favorable adsorption (*n* = 2.8) with high adsorption capacity (KF = 118 ((mg g⁻¹) (L mg⁻¹) ^(1/n)^), despite moderate goodness-of-fit (R² = 0.818), suggesting heterogeneous adsorption sites, favorable adsorption conditions and strong adsorbent–adsorbate interactions^53,54^. However, its lowest R² (0.818) among the three models indicates poor conformity to its assumptions^55^. The Temkin isotherm provided better fit (R² = 0.895) than Freundlich, indicating uniform adsorption energy (B = 167 J/mol) and moderate binding strength (K_T_ = 0.367 L/mg) and moderate interaction strength between the adsorbate and adsorbent, that may suggest equal distribution of binding energies. Although its R² value of 0.895 is higher than that of the Freundlich model, it remains lower than that of the Langmuir model, indicating that Temkin provides a reasonable but less accurate description of the adsorption process^56,57^.

**Table 2:** Fitting parameters of Freundlich, Langmuir, and Temkin models for COD removal from greywater.

Isothermal model	Parameter	Greywater
Freundlich	K_f_ ((mg g⁻¹) (L mg⁻¹) ^(1/n)^)	114
	n	2.80
	R^2^	0.812
Langmuir	q_m_ (mg/g)	722
	K_L_ (L/mg)	0.045
	R^2^	0.924
Temkin	B (J mol⁻¹)	167
	K_T_ (L/mg)	0.367
	R^2^	0.895

To evaluate the adsorption kinetics of greywater treatment, the experimental results were fitted using pseudo-first-order (PFO) and pseudo-second-order (PSO) kinetic models. The constants and data resulted from kinetic models are shown in Table 3. Both models yielded high R² values (0.987 for PFO and 0.988 for PSO), indicating that each provides an acceptable description of the adsorption rate. Nevertheless, the nonlinear PSO model offered slightly better accuracy, as evidenced by its higher adjusted R² (0.987) and lower reduced chi-square value (189) relative to the PFO model. For the PSO model, the equilibrium adsorption capacity (qₑ) was determined to be 472 mg/g, which is in close agreement with the experimental data, while the rate constant (k_2_) was 4.80 × 10⁻⁵ g/mg·min. This strong consistency supports that adsorption occurs chemically (chemisorption), involving valence forces associated with electron sharing or exchange between UiO-66 and the organic pollutants present in greywater^58^.

In comparison, the PFO model produced a value of 381 mg/g and rate constant b = 0.022 min⁻¹, but a slightly higher chi-square (201), indicating marginally weaker predictive capacity. Together, these findings confirm that the adsorption process follows pseudo-second-order kinetics and Langmuir isotherm behavior, suggesting monolayer chemisorption on a homogeneous surface with high active site availability.

**Table 3:** Kinetic model parameters for greywater adsorption onto UiO-66 1:0.75, including rate constants, equilibrium capacities, and statistical fit indicators for PSO and PFO models.

Model	Parameters				
PFO	qe (mg/g)	k_1_ (min^− 1^)	R^2^	Reduced Chi-Sqr	Adj. R^2^
	381 ± 8.8	0.021 ± 0.002	0.987	201	0.986
PSO	qe (mg/g)	K_2_ (g mg^− 1^ min^− 1^)	R^2^	Reduced Chi-Sqr	Adj. R^2^
	472 ± 17.71	4.79E-5 ± 7.73E-6	0.988	189	0.987

### Regeneration study and leaching possibility

The potential to regenerate and reuse adsorbents holds significant environmental and economic importance. Repeated use of adsorbents reduces the need for continuous production of new materials, thereby minimizing waste generation and resource consumption. To evaluate the reusability of the adsorbent, three consecutive adsorption-desorption cycles were conducted. In each cycle, following the completion of the adsorption process, the adsorbent was washed with 0.1 M NaOH solution followed by ethanol. The maximum equilibrium adsorption capacities derived from the Langmuir isotherm for each cycle are presented in Table 4. The results revealed adsorption capacities of 722 mg/g (1ˢᵗ cycle), 640 mg/g (2ⁿᵈ cycle), and 570 mg/g (3ʳᵈ cycle), demonstrating considerable retention of adsorption capacity despite the gradual decline. To verify UiO-66 structural integrity under optimized conditions and absence of Zr leaching, Graphite furnace atomic absorption spectrometry (GFAAS) analysis was performed and results showed zirconium leaching of 0.009 mg/L, confirming the excellent structural stability of UiO-66.

**Table 4:** regeneration study of UiO-66 by evaluating maximum equilibrium adsorption capacities derived from the Langmuir isotherm.

Parameter	1st adsorption cycle	2nd adsorption cycle	3rd adsorption cycle
q_m_ (mg g^− 1^)	722	640	570

### Comparison study

A comparative evaluation of the synthesized UiO-66 1:0.75 for COD removal from greywater is presented in Table S6. Since, UiO-66 has not been used for greywater adsorption based on the conducted reviews, comparisons with other adsorbents are reported here. The results indicate that UiO-66 1:0.75 achieved a removal efficiency of 88.4% at pH 2 within 3 h, which is higher than those of the industrial activated carbon used in this study (67.3%, pH 5.5, 3 h), biologically active GAC (71%, natural pH, 24 h)^59^, inhibited BAC (64%, natural pH, 24 h)^59^, zeolite (40%, natural pH, 3 h)^60^, conventional activated carbon (57%, natural pH, 3 h)^60^, and nano zero-valent iron (60%, natural pH, 3 h)^60^. An important observation from this comparison is the relatively shorter contact time required for UiO-66 1:0.75 to achieve higher removal efficiency. While some adsorbents such as biologically active GAC exhibit moderate performance, they require significantly longer treatment durations (24 h), whereas the present material reaches a higher efficiency within only 3 h. Additionally, when compared under similar contact times (3 h), UiO-66 1:0.75 consistently outperforms conventional adsorbents including activated carbon, zeolite, and nano zero-valent iron, indicating more efficient utilization of adsorption sites and improved interaction with greywater contaminants. It should be noted, however, that direct comparison between studies must be interpreted with caution due to differences in experimental conditions, particularly pH. The superior performance of UiO-66 1:0.75 was obtained under strongly acidic conditions, whereas most of the other adsorbents were evaluated at natural pH. Despite this limitation, the overall comparison suggests that the optimized UiO-66 adsorbent exhibits good and comparatively superior performance for COD removal from greywater. These findings support the effectiveness of the material and also highlight the potential of controlled MOF synthesis as a promising strategy for developing efficient adsorbents for wastewater treatment applications.

## Conclusion

This study evaluated the potential of UiO-66 metal–organic framework adsorbents for greywater treatment and examined the influence of synthesis conditions and operational parameters on adsorption performance. UiO-66 materials synthesized with different metal-to-ligand molar ratios of zirconium tetrachloride to terephthalic acid were characterized using XRD, BET, FTIR, SEM, and EDS analyses. Among the synthesized materials, UiO-66 1:0.75 exhibited improved crystallinity, a higher surface area (1387 m² g⁻¹), and a larger pore volume (1.66 cm³ g⁻¹), indicating favorable structural characteristics for adsorption applications. Batch adsorption experiments showed that the removal efficiency of greywater pollutants was significantly influenced by operational parameters, namely initial COD concentration, adsorbent dosage, pH, and contact time. Kinetic analysis indicated that adsorption reached equilibrium after approximately 3 h, suggesting relatively rapid pollutant uptake by the UiO-66. Kinetic analysis also showed that the adsorption process followed the pseudo-second-order model, indicating that surface interactions between the adsorbent and organic pollutants play an important role in the adsorption mechanism. Equilibrium analysis demonstrated that the Langmuir isotherm provided the best fit to the experimental data, suggesting monolayer adsorption on a relatively homogeneous surface. Using the ANN–genetic algorithm approach, optimal operating conditions were identified (COD₀ = 236 ppm, adsorbent dosage = 500 ppm, pH = 2, and contact time = 3 h), under which the UiO-66 1:0.75 adsorbent achieved a maximum COD removal efficiency of 88.4%. The ANN model demonstrated high predictive capability for analyzing the influence of operational parameters and identifying optimal treatment conditions, providing insight into the nonlinear relationships between system variables and adsorption efficiency.

Despite these promising results, several limitations should be acknowledged. The experiments were conducted using synthetic greywater under controlled laboratory conditions, which may not fully represent the chemical complexity and variability of real domestic greywater. In addition, the optimal pH identified in this study is strongly acidic, which may require further evaluation for practical implementation in full-scale treatment systems. Furthermore, thermodynamic analysis was not performed, which limits a deeper understanding of the adsorption spontaneity and its energetic characteristics. Future studies should therefore focus on evaluating adsorption performance using real greywater matrices, investigating long-term stability, performing thermodynamic analyses, and assessing process scalability and operational feasibility for practical wastewater treatment applications. Overall, the findings demonstrate that controlled synthesis of UiO-66 combined with data-driven process optimization provides a promising framework for improving MOF-based adsorption systems for greywater treatment.

## Supplementary Information

Below is the link to the electronic supplementary material.

Supplementary Material 1

## Data Availability

All data generated or analyzed during this study are included in this published article [and its Supplementary Information files].
