# Supplementary material for: Optimization of greywater treatment using UiO-66 nanomaterial: artificial neural network modeling
Source: Sci Rep. 2026 Apr 13;16:17199. doi: 10.1038/s41598-026-48174-2 (PMC13233985; doi:10.1038/s41598-026-48174-2)
Supplement: Supplementary file 1 — Supplementary Material 1 [file 41598_2026_48174_MOESM1_ESM.docx]

**Optimization of Greywater Treatment using UiO-66 Nanomaterial: Artificial Neural Network Modeling**

Aliakbar Zare^1^, Mehrzad Feilizadeh^1,^^[[1]](#footnote-1)^, Zahra Derakhshan^2^, Jamal rasouli^1^

*^1^* *Department of Chemical Engineering, School of Chemical and Petroleum Engineering, Shiraz University, Shiraz, Iran*

*^2^ Department of Environmental Health Engineering, School of Public Health, Shiraz University of Medical Sciences, Shiraz, Iran*

# Materials

Zirconium (IV) chloride (ZrCl_4_), terephthalic acid (C_8_H_6_O_4_), dimethylformamide (C_3_H_7_NO), were obtained from Merck company. COD digestion vials were obtained from Hach company. Absolute ethanol (C₂H₆O, 99.8%) was supplied by Zakaria Jahrom Company. Additionally, industrial activated carbon (surface area = 554 m^2^) was obtained from Xinyou Aquarium Product Factory. It is worth mention that none of the materials used required further purification because they were all of analytical quality.

# Synthetic greywater composition

To prepare greywater with a specific COD, first, different components were added to 1000 mL of water according to Chrispim and Nolasco [1] with a slight modification (Table S1) in a specified mass or volume. Then, the dilution was done by adding appropriate amount of deionized water to achieve the target COD value.

Table S1: Various components used for greywater preparation.

| Component | Content |
| --- | --- |
| Shampoo | 4.7 g |
| Body lotion | 8.5 g |
| Toothpaste | 1.2 g |
| Soap | 6 g |
| Laundry detergent | 7 ml |
| Laundry softener | 5 ml |
| Hair softener | 4.8 g |
| Sun screen | 1 g |
| Lactic acid | 1 ml |
| NaHCO_3_ | 0.45 g |
| Na_2_PO_4_ | 0.81 g |
| Na_2_SO_4_ | 0.81 g |

# Adsorption calculations

The adsorption performance of the synthesized UiO-66 adsorbent was evaluated using removal efficiency and adsorption capacity calculations. Removal efficiency (%) was determined to quantify the extent of organic pollutant removal from greywater, while adsorption capacity (qₑ) was used to describe the amount of contaminant adsorbed per unit mass of adsorbent. Removal efficiency was calculated using the following equation:

| $Removal Efficiency \left( \% \right)=\frac{\left( C_{0}-C_{e} \right)}{C_{0}}*100$ | (1) |
| --- | --- |

In this equation, C₀ (ppm) is the initial COD concentration and Cₑ (ppm) is the equilibrium COD concentration after adsorption. The adsorption capacity at equilibrium (qₑ) was calculated as

| $q_{e}=\frac{\left( C_{0}-C_{e} \right)*V}{m}$ | (2) |
| --- | --- |

where qₑ (mg g⁻¹) represents the equilibrium adsorption capacity, V (L) is the volume of the solution, and m (g) is the mass of the adsorbent used in the experiment.

# Interpretation of 3D Plots

Figure 6a illustrates the effects of adsorbent concentration and COD_0_​ on removal efficiency. The results demonstrate a direct correlation between adsorbent concentration and efficiency, with higher adsorbent concentration​ leading to improved pollutant removal. This enhancement can be attributed to the increased number of active adsorption sites available for binding contaminants at higher adsorbent dosages. However, the efficiency plateaus at high adsorbent concentrations because the available adsorption sites exceed the number of pollutant molecules, and particle aggregation may reduce effective surface area, leading to diminished adsorption efficiency per unit mass. Additionally, the influence of COD_0_​ is apparent, as lower COD_0_ values yield higher removal efficiencies. This trend is likely driven by the reduced competition among contaminants for active adsorption sites when the pollutant load is lower.

Figure 6b illustrates the combined effects of pH and COD₀ on greywater removal efficiency. A clear dependence of adsorption performance on pH is observed, with acidic conditions resulting in significantly higher removal efficiencies. However, because greywater COD represents a complex mixture of organic compounds—including neutral, weakly ionizable, and non-ionic species—the adsorption mechanism cannot be attributed solely to electrostatic interactions. Instead, the pH dependence likely reflects a combination of surface charge effects and changes in the chemical environment at the adsorbent–solution interface [2].

To better understand the role of surface charge, the point of zero charge (pHpzc) of the UiO-66 adsorbent was determined (Figure S5a), yielding a value of approximately 6.4. This result indicates that the surface of UiO-66 becomes positively charged at pH values below 6.4 and negatively charged at higher pH values. Under acidic conditions, the protonation of surface hydroxyl groups associated with zirconium nodes may enhance interactions between the MOF framework and certain polar or negatively charged organic species present in greywater. However, since COD reflects the total concentration of oxidizable organic compounds rather than a single ionic species, additional adsorption mechanisms such as pore filling, hydrogen bonding, π–π interactions between aromatic linkers and organic molecules, and van der Waals interactions may also contribute to pollutant removal.

Figure 6c illustrates the combined effects of time and COD_0_ on removal efficiency. The 3D surface plot demonstrates that removal efficiency initially increases with time, reaching a plateau as equilibrium is approached. This behavior is attributed to the progressive occupation of active adsorption sites on the UiO-66 1:0.75 surface. At the start of the adsorption process, the high concentration gradient between the solution and adsorbent surface drives rapid pollutant uptake. As equilibrium nears, this gradient diminishes, leading to slower adsorption rates. This reduction is likely due to the saturation of adsorption sites by a higher pollutant load, limiting the capacity of the adsorbent. The trends observed in Figure 6d further supports the combined influence of pH and adsorbent concentration on adsorption efficiency. Increasing adsorbent dosage improves removal efficiency due to the higher availability of active adsorption sites and increased accessible surface area. Acidic conditions further enhance performance, although this improvement should be interpreted as the result of multiple concurrent adsorption mechanisms rather than purely electrostatic attraction [3].

Figure 6e examines the effects of time and adsorbent concentration ​ on removal efficiency. The 3D surface plot reveals a significant increase in removal efficiency with longer adsorption times and higher adsorbent concentrations. At shorter time intervals, the efficiency remains low, regardless of the adsorbent concentration, indicating that insufficient time limits pollutant diffusion into the adsorbent’s active sites. As time progresses, the efficiency rises steeply, particularly at higher adsorbent concentrations, where the increased number of active sites accelerates pollutant uptake. The contour plot further confirms this trend, with higher efficiencies observed at extended adsorption times and elevated adsorbent concentration​, reflecting a synergistic enhancement in adsorption performance. This behavior is consistent with the kinetics of adsorption, where time is critical for approaching equilibrium, and a higher adsorbent dosage provides additional adsorption capacity.

Similarly, Figure 6f demonstrates the combined effects of pH and contact time. Longer contact times allow sufficient diffusion of organic molecules into the porous structure of UiO-66, promoting adsorption until equilibrium is approached. While acidic pH values lead to higher removal efficiencies, this effect likely arises from the combined influence of surface charge behavior, enhanced molecular interactions within the MOF pores, and changes in pollutant speciation in solution. Overall, these results suggest that pH plays an important role in modulating the adsorption environment, although the removal of greywater COD by UiO-66 is governed by a combination of electrostatic and non-electrostatic interactions.


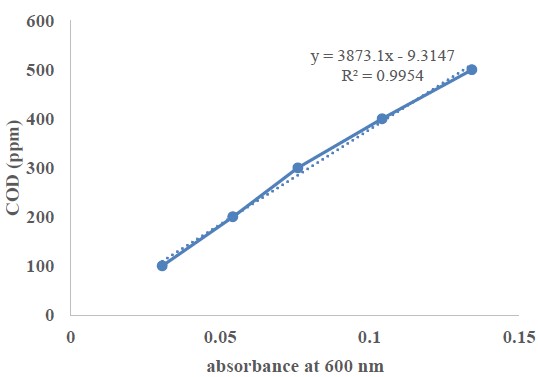


Figure S1. COD standard curve used in this study.


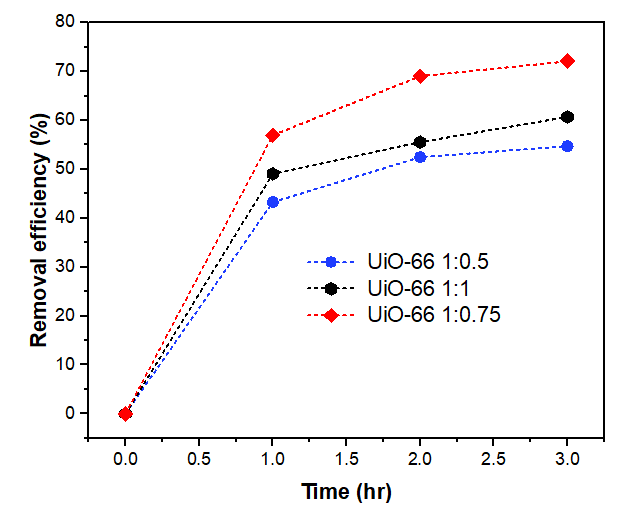


Figure S2. Comparison of removal efficiencies of UiO-66 variants with ligand-to-metal ratios of 1:1, 1:0.75, and 1:0.5.


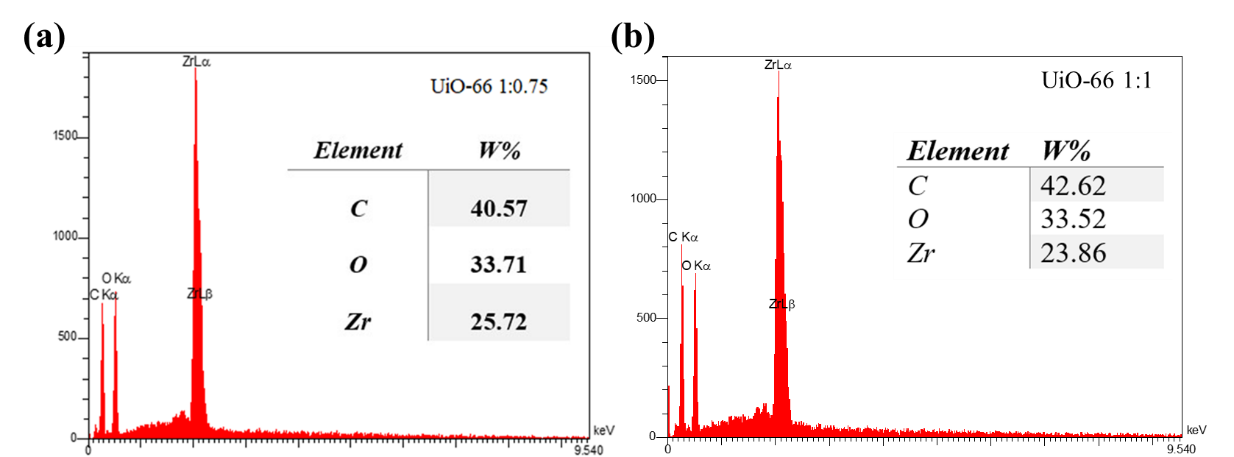


Figure S3. EDS spectra of a) UiO-66 1:0.75, and b) UiO-66 1:1.


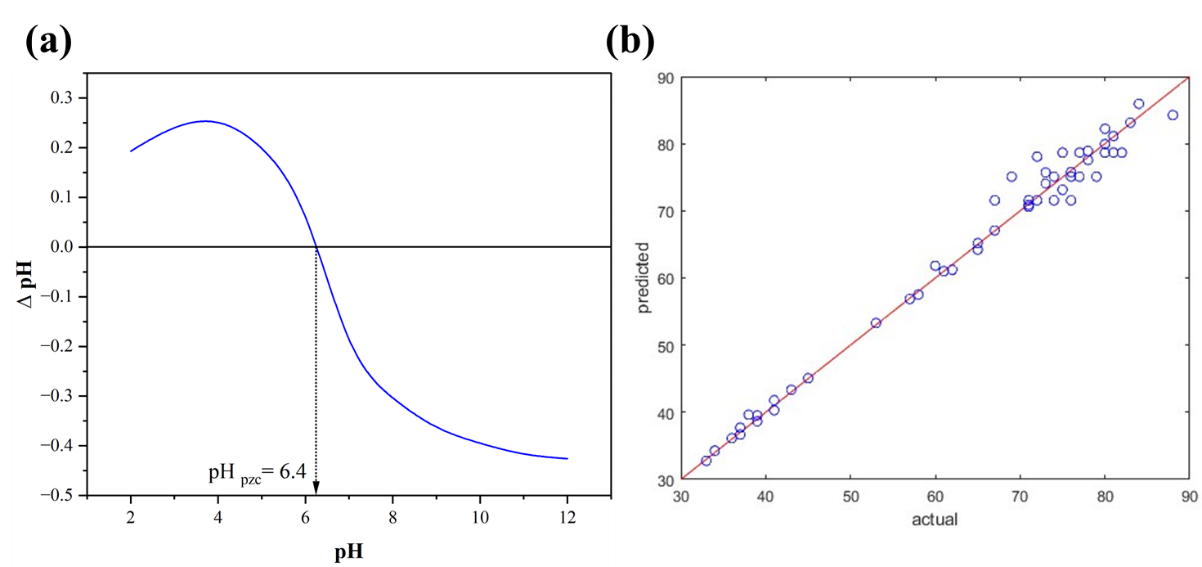


Figure S4. a) point of zero charge for UiO-66, b) comparison of experimental data with ANN model predictions.


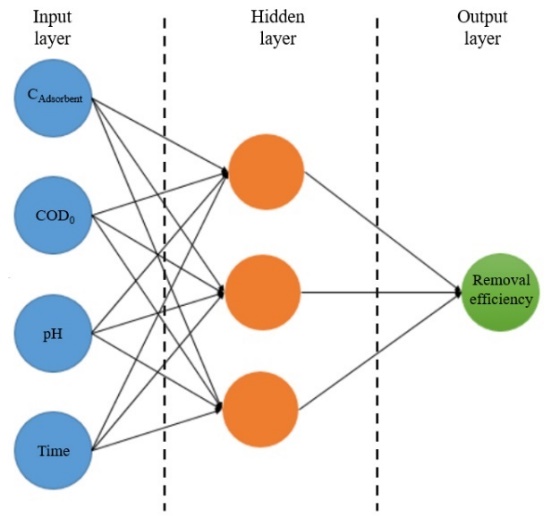


Figure S5. Schematic of the artificial neural network used.

Table S2. Arrangement of the BBD and ANN for the four independent variables and the greywater removal efficiency.

| Run. | COD_0_ | C- Adsorbent | pH | Time (h) | Removal (%) | Removal (%) |
| --- | --- | --- | --- | --- | --- | --- |
|  |  |  |  |  | **Experimental** | **ANN** |
| 1 | 300 | 100 | 7 | 1 | 34.0 | 34.3 |
| 2 | 300 | 100 | 2 | 1 | 39.0 | 38.7 |
| 3 | 200 | 300 | 2 | 1 | 73.0 | 75.7 |
| 4 | 300 | 500 | 2 | 1 | 80.3 | 79.9 |
| 5 | 200 | 100 | 4.5 | 1 | 37.0 | 37.7 |
| 6 | 300 | 300 | 4.5 | 1 | 72.0 | 71.6 |
| 7 | 400 | 500 | 4.5 | 1 | 61.0 | 61.0 |
| 8 | 200 | 300 | 7 | 1 | 70.5 | 70.9 |
| 9 | 200 | 500 | 4.5 | 1 | 71.5 | 78.1 |
| 10 | 300 | 500 | 7 | 1 | 71.3 | 70.6 |
| 11 | 400 | 100 | 4.5 | 1 | 33.3 | 32.8 |
| 12 | 400 | 300 | 7 | 1 | 53.0 | 53.3 |
| 13 | 300 | 300 | 4.5 | 1 | 71.0 | 71.6 |
| 14 | 400 | 300 | 2 | 1 | 56.8 | 56.9 |
| 15 | 300 | 300 | 4.5 | 1 | 67.0 | 71.6 |
| 16 | 300 | 300 | 4.5 | 1 | 76.0 | 71.6 |
| 17 | 300 | 300 | 4.5 | 1 | 74.0 | 71.6 |
| 18 | 300 | 100 | 7 | 2 | 37.3 | 36.7 |
| 19 | 300 | 100 | 2 | 2 | 41.0 | 41.8 |
| 20 | 200 | 300 | 2 | 2 | 78.0 | 78.9 |
| 21 | 300 | 500 | 2 | 2 | 83.0 | 83.1 |
| 22 | 200 | 100 | 4.5 | 2 | 40.5 | 40.3 |
| 23 | 300 | 300 | 4.5 | 2 | 76.0 | 75.1 |
| 24 | 400 | 500 | 4.5 | 2 | 64.8 | 64.2 |
| 25 | 200 | 300 | 7 | 2 | 74.5 | 73.1 |
| 26 | 200 | 500 | 4.5 | 2 | 81.0 | 81.1 |
| 27 | 300 | 500 | 7 | 2 | 73.0 | 74.1 |
| 28 | 400 | 100 | 4.5 | 2 | 35.5 | 36.1 |
| 29 | 400 | 300 | 7 | 2 | 58.0 | 57.5 |
| 30 | 300 | 300 | 4.5 | 2 | 74.0 | 75.1 |
| 31 | 400 | 300 | 2 | 2 | 61.5 | 61.2 |
| 32 | 300 | 300 | 4.5 | 2 | 69.0 | 75.1 |
| 33 | 300 | 300 | 4.5 | 2 | 79.0 | 75.1 |
| 34 | 300 | 300 | 4.5 | 2 | 77.0 | 75.1 |
| 35 | 300 | 100 | 7 | 3 | 39.0 | 39.5 |
| 36 | 300 | 100 | 2 | 3 | 45.3 | 45.1 |
| 37 | 200 | 300 | 2 | 3 | 79.5 | 82.2 |
| 38 | 300 | 500 | 2 | 3 | 84.0 | 85.9 |
| 39 | 200 | 100 | 4.5 | 3 | 42.5 | 43.3 |
| 40 | 300 | 300 | 4.5 | 3 | 80.0 | 78.7 |
| 41 | 400 | 500 | 4.5 | 3 | 67.0 | 67.1 |
| 42 | 200 | 300 | 7 | 3 | 76.0 | 75.8 |
| 43 | 200 | 500 | 4.5 | 3 | 87.5 | 84.3 |
| 44 | 300 | 500 | 7 | 3 | 78.0 | 77.6 |
| 45 | 400 | 100 | 4.5 | 3 | 37.8 | 39.6 |
| 46 | 400 | 300 | 7 | 3 | 59.8 | 61.8 |
| 47 | 300 | 300 | 4.5 | 3 | 77.0 | 78.7 |
| 48 | 400 | 300 | 2 | 3 | 65.0 | 65.2 |
| 49 | 300 | 300 | 4.5 | 3 | 75.0 | 78.7 |
| 50 | 300 | 300 | 4.5 | 3 | 82.0 | 78.7 |
| 51 | 300 | 300 | 4.5 | 3 | 81.0 | 78.7 |

Table S3. Statistical parameters of the ANN model.

| parameters | results |
| --- | --- |
| R^2^ all data | 0.983 |
| R^2^ adjust | 0.982 |
| RMSE | 2.117 |
| MSE | 4.480 |
| MAE | 1.436 |
| AAD | 2.130 |
| R^2^ train | 0.986 |
| R^2^ validation | 0.979 |
| R^2^ test | 0.971 |

Table S4. Optimum conditions of operational parameters for greywater COD removal.

| Parameter | Amount |
| --- | --- |
| COD concentration | 236 ppm |
| C- Adsorbent | 500 ppm |
| pH | 2 |
| Time | 3h |
| Removal | 88.4% |

Table S5. relative importance of each input parameter according to Garson’s algorithm.

| Parameter | relative importance (%) |
| --- | --- |
| COD concentration | 33 |
| C- Adsorbent | 47.9 |
| pH | 11.5 |
| Time | 7.6 |

Table S6. Comparison of adsorption performance of UiO-66 1:0.75 with other adsorbents for COD removal from greywater

| Adsorbent | Pollutant  (evaluated parameter) | Removal efficiency (%) | Key conditions | Reference |
| --- | --- | --- | --- | --- |
| UiO-66 (1:0.75) | Greywater (COD) | 88.4 | pH 2, 3 h | This study |
| Industrial Activated Carbon | Greywater (COD) | 67.3 | pH 5.5, 3 h | This study |
| Biologically active GAC (BAC) | Greywater (COD) | 71 | Natural pH, 24 h | [4] |
| Inhibited BAC (InBAC) | Greywater (COD) | 64 | Natural pH, 24 h | [4] |
| Zeolite | Greywater (COD) | 40 | Natural pH, 3 h | [5] |
| Activated carbon | Greywater (COD) | 57 | Natural pH, 3 h | [5] |
| Nano zero valent iron | Greywater (COD) | 60 | Natural pH, 3 h | [5] |

**References**

1. M.C. Chrispim, M.A.N., Greywater treatment using a moving bed biofilm reactor at a university campus in Brazil. Journal of cleaner production, 2017. 142: p. 290–296.

2. Rajabi, S., et al., *Innovative grey water treatment using eco-friendly bio-photocatalyst AgCuFe_2_O_4_@chitosan in the presence of synergistic effects of persulfate activation: optimization and mechanisms.* International Journal of Biological Macromolecules, 2025. 286: p. 138375.

3. Rajabi, S., et al., *Synergistic degradation of metronidazole and penicillin G in aqueous solutions using AgZnFe_2_O_4_@chitosan nano-photocatalyst under UV/persulfate activation.* Environmental Technology & Innovation, 2024. 35: p. 103724.

4. Sharaf, A. and Y. Liu, *Mechanisms and kinetics of greywater treatment using biologically active granular activated carbon.* Chemosphere, 2021. 263: p. 128113.

5. Amiri, M.J., et al., *Greywater treatment using single and combined adsorbents for landscape irrigation.* Environmental Processes, 2019. 6(1): p. 43-63.

1. **Corresponding authors:** Mehrzad Feilizadeh E-mail: [m.feilizadeh@shirazu.ac.ir](mailto:m.feilizadeh@shirazu.ac.ir) [↑](#footnote-ref-1)
